# Turning Waste into Treasure: Invasive Plant *Ambrosia trifida L* Leaves as a High-Efficiency Inhibitor for Steel in Simulated Pickling Solutions

**DOI:** 10.3390/ma17153758

**Published:** 2024-07-30

**Authors:** Xin Sun, Huiwen Tian, Fangxin Zou, Weihua Li, Yujie Qiang, Baorong Hou

**Affiliations:** 1 Key Laboratory of Advanced Marine Materials, Key Laboratory of Marine Environmental Corrosion and Bio-Fouling, Institute of Oceanology, Chinese Academy of Sciences, Qingdao 266071, Chinabaoronghou@163.com (B.H.); 2 Department of Aeronautical and Aviation Engineering, The Hong Kong Polytechnic University, Hung Hom, Kowloon, Hong Kong SAR, China; frank.zou@polyu.edu.hk; 3 Henan Academy of Sciences, Zhengzhou 450046, China; liweihua1928@163.com; 4 North China University of Water Resources and Electric Power, Zhengzhou 450046, China; 5 National Center for Materials Service Safety, University of Science and Technology Beijing, Beijing 100083, China

**Keywords:** plant extract, corrosion, Q235 steel, theoretical calculation, electrochemistry

## Abstract

High toxicity is the main reason for the limited application of traditional corrosion inhibitors. Herein, it is critical to find a green, efficient, and long-term stable alternative substitute for the hazardous and conventional corrosion inhibitor. *Ambrosia trifida L* is widely distributed in fields and riverside wetlands as an invasive plant in China. According to the concept of turning waste into treasure, the extract of *Ambrosia trifida L* leaves (ATL) has the potential to address this issue due to its natural origin and abundant presence of heterocyclic organics. Therefore, ATL, as a green corrosion inhibitor, is prepared for the first time via a simple water-based extraction method. FT-IR (Fourier transform infrared spectroscopy) and UV-Vis (UV-visible) indicate that ATL extract contains abundant heterocyclic organics with conjugated structures, which exhibit the potential to become a high-efficiency inhibitor. Notably, the active sites of ATL molecules and their interaction with Q235 steel at the molecular/atomic level are revealed via theoretical calculations. The highest *E*_binding_ value observed for the major components in the ATL extract is 259.66 kcal/mol, implying a significant adsorption capacity. The electrochemical results verify that microdose ATL extract can prominently inhibit steel corrosion, and the highest inhibition efficiency (*η*) is 97.5% (1000 mg/L). Following immersion for 24 h, the *η* value is enhanced to 99.0%, indicating a reliable and long-term ATL extract protection film is formed on the steel surface in harsh acidic solutions. The results of the weight loss, SEM (scanning electron microscope), and LSCM (laser scanning confocal microscopy) are consistent with the above conclusions. Finally, this study anticipates providing theoretical support for developing novel green plant extract inhibitors and aiding in their application in industrial pickling environments.

## 1. Introduction

Steel products are extensively utilized daily because of their exceptional physical/chemical qualities, cost-effective manufacture, and vast reservoirs of resources [1,2,3]. Among them, Q235 steel, renowned for its commendable plasticity and welding property, finds application in oil and gas delivery, the chemical industry, structural engineering, and machine manufacturing [4]. However, unlike other corrosion-resistant metals (noble metal and stainless steel), Q235 steel is easily corroded in the environment, restricting further processing. In moist environments, Q235 steel substrate can rapidly absorb and accumulate small quantities of water, forming a thin liquid film. Then, active molecules (O_2_ and CO_2_) continuously dissolve in the liquid layer, creating an aggressively corrosive solution. Finally, an oxygen absorption reaction occurs on the steel surface, which further accumulates corrosion products (Fe_2_O_3_, Fe(OH)_3,_ and others) [5]. The mechanical properties of service steel-based equipment, such as strength, toughness, and plasticity, deteriorate due to corrosion degradation, which may cause safety problems [6,7,8]. With the occurrence of accidents, the economy and environment will suffer significant losses [9]. As a practical surface treatment technology, pickling can effectively remove corrosion products from metal surfaces to ensure their performance stability. Notably, pickling solutions can inevitably erode steel substrates, leading to structural damage and higher processing costs. The introduction of microdose corrosion inhibitors can solve the above problems to a great extent [10]. Over the past few decades, inorganic corrosion inhibitors have been routinely employed to decrease metal corrosion rates in acidic environments. However, the high toxicity of inorganic inhibitors (Cr^3+^/Cr^6+^ and NO_2_^−^) restricts their sustainable application [11]. Hence, inorganic inhibitors are gradually being abandoned or replaced by organic inhibitors.

Numerous studies have shown that the inhibition performance of organics usually comes from heteroatoms (N, O, S, and P), unsaturated chemical bonds (C=C/C≡C), and conjugated structures (benzene ring). Based on this, organic inhibitors, represented by azole [12], amino acids [13], and ionic liquid derivatives [14], have been abundantly exploited. At the same time, researchers also attempt to reveal the interaction between inhibitors and the metal interface via experimental and theoretical calculations. Qiang et al. found that 5-(Benzylthio)-1H-tetrazole (BTTA) could provide credible protection for steel corrosion in HCl solution, and the highest protection efficiency reaches up to 97.7% [15]. The protection mechanism originated from the multiple anchoring interactions of BTTA molecules on metal surfaces, as further demonstrated by molecular modeling. Pour-Ali et al. explored the corrosion inhibition of three L-amino acids (L-asparagine, L-isoleucine, and L-proline) for steel in 0.5 M HCl [16]. The results showed that L-asparagine (255.3 kJ/mol) exhibited optimal inhibition performance (~95%) due to the higher binding energy when compared with other L-amino acids (163.2 and 125.5 kJ/mol). A bio-based ionic liquid was synthesized by Aslam et al., and its corrosion inhibition for steel in different-temperature HCl solutions was evaluated [17]. Although organic corrosion inhibitors exhibit satisfying steel protective properties in acidic environments, some specific problems still need to be considered, including complex synthesis steps, solubleness in water, and elevated expenses. Therefore, there is great scientific and practical significance in developing corrosion inhibitors with easy fabrication methods, good water solubility, and low cost.

Plant extract inhibitors have been widely investigated recently because of their incomparable advantages, such as being green, degradable, readily available, renewable, and simple to prepare [18]. Meanwhile, plant extracts exhibit potential as efficient corrosion inhibitors with a diverse range of heterocyclic organic compounds (flavonoids, quinonoids, coumarins, and others) [19]. Based on macro- and micro-electrochemical techniques, Li et al. simultaneously proved the protection performance of Brassica oleracea L for steel in HCl and H_2_SO_4_ solutions [20]. The theoretical calculations found that heterocyclic organic compounds can form a dense film on the metal surface via parallel adsorption. According to electrochemical impedance spectroscopy (EIS) and micromorphological characteristics, Qiang et al. certified the protection effect of *platanus acerifolia leaf* extract (PAL) on steel in HCl solution [21]. With the rise in experimental temperature, PAL still processed dependable corrosion inhibition performance (93.1% at 308 K and 87.8% at 318 K). Liao et al. analyzed the chemical composition of the fructus cannabis protein extract (FP) by FT-IR, UV-Vis, and LC/MS [22]. Then, the authors found that 100 mg/L FP exhibited the highest protection efficiency (97.9%), which is also optimal compared to similar work. Many researchers have demonstrated the corrosion protection of plant extracts for steel in acidic environments [23]. Furthermore, the impact of temperature on plant extract inhibition performance has also been considered [24]. However, researchers have not given much consideration to the long-term corrosion protection properties of plant extracts on metals. Without a doubt, long-term corrosion resistance is essential for the industrial applications of plant extracts, particularly in cost and quality control. Furthermore, it’s still indispensable to comprehensively investigate the interface interaction mechanism between plant extract molecules and steel substrate, which is beneficial to the development and industrial application of novel plant extract inhibitors.

*Ambrosia trifida L* is widely distributed in fields and riverside wetlands as an invasive plant in China, harming wheat, soybeans, and various horticultural crops. Considering its various natural phenolic acid organics (syringic acid, quercetin, chlorogenic acid, and others), *Ambrosia trifida L* has the potential to become an efficient corrosion inhibitor [25]. This work prepared *Ambrosia trifida L* leaf extract (ATL) via a water-based extraction method to ensure excellent solubility in acid solutions. The adsorption behavior and interaction mechanism at the ATL extract/steel substrate interface were revealed through molecular modeling. The impact of ATL concentration and immersion time on steel corrosion protection was assessed based on weight loss and electrochemical tests. Then, the morphology change of steel under the protection of ATL was explored via SEM and LSCM. This work aims to provide insights into the development of novel plant extract inhibitors and promote their practical application in industrial pickling environments.

## 2. Experimental

### 2.1. Materials and Solution

Concentrated hydrochloric acid (~37 wt.%) and other reagents were provided by China Sinopharm Group. Q235 steel was purchased from the China Shengxin corrosion sample center. The corrosive solution was 1 M HCl, produced by concentrated hydrochloric acid and deionized water (18.25 MΩ cm). The conventional chemical reagents used in the experiment were purchased from China Shanghai Aladdin Biochemical Technology Co., Ltd. (Shanghai, China). In addition, the added dose of ATL extract was 0, 50, 100, 200, and 1000 mg/L.

### 2.2. ATL Extract Preparation

**Figure 1:**
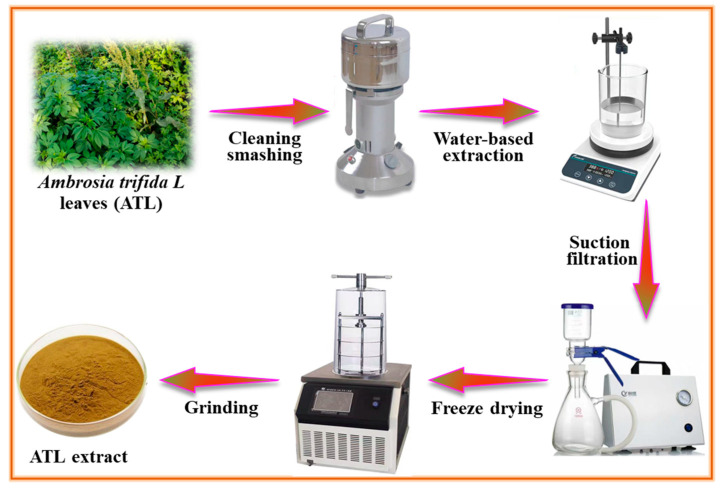
The preparation schematic diagram of the ATL extract.

The fresh *Ambrosia trifida L* leaves were collected near the Institute of Oceanology in China. The extraction technique followed a similar process described by Li et al. [20]. As shown in Figure 1, fresh ATL was rinsed with deionized water to remove pollutants and grime. After that, the ATL was dried at 80 ℃ for 24 h and then crushed via a pulverizer. Subsequently, boiling deionized water was used to extract the main ingredients of ATL powder. Following a 24 h extraction process, a negative pressure filtration device separated the brown mixture, and the resulting clear filtrate was subjected to freeze-drying for 48 h. After being ground using an agate mortar, the dark yellow ATL extract powder was finally gathered. The main functional group characteristics of ATL were analyzed via FT-IR (Nicolet 6700, Thermo Scientific, Waltham, MA, USA) and UV-Vis (Lambda 950, Perkin Elmer Limited, Waltham, MA, USA). The FT-IR test was conducted from 4000 to 400 cm^−1^, and the resolution was 4 cm^−1^. For the UV-Vis, the scan range and speed were 400 to 200 nm and 1 nm/s, respectively.

### 2.3. Electrochemical Tests

The inhibition performance of the ATL extract for Q235 steel in acidic solutions was accessed through the conventional three-electrode test system, which contained a working electrode (1 × 1 cm^2^), a counter electrode (Pt sheet 2 × 2 cm^2^), and a reference electrode (a saturated calomel electrode). To accurately detect small changes in the working electrode during the test, copper wire was fixed to the steel surface with electric welding. Except for the working surface, the other sides were sealed with epoxy resin to isolate the contact of corrosive mediums. The counter and reference electrodes were purchased from China Tianjin Aida Hengsheng Technology Development Co., Ltd. (Tianjin, China). All electrochemical experiments were conducted on a CHI 760e (CH Instruments, Inc., Shanghai, China). The OCP (open circuit potential) was employed to monitor the stability of the test system. The EIS test was operated based on the stable OCP value, in which the test frequency and disturbance signal were 10^5^ to 10^−2^ Hz and 5 mV, respectively. There were 12 sampling points for each frequency interval. The immersing time was 1200 s when evaluating the effect of ATL concentration on the corrosion protection of steel in 1 M HCl solution, while the immersion periods for long-term corrosion inhibition were 2, 12, and 24 h. The inhibition efficiency (*η*_EIS_) of ATL extract was obtained as follows:

(1) $$\eta_{\mathrm{EIS}}(\%)=\frac{R_{\mathrm{Sum}}-R_{\mathrm{Sum},0}}{R_{\mathrm{Sum}}}\times 100$$

where *R*_Sum_ and *R*_Sum,0_ represent the summation resistance (*R*_f_ + *R*_ct_) of steel with and without ATL extract, respectively. For the polarization measurement, the initial and final scanning potential values were −250 mV and +250 mV based on the OCP value, and the scanning rate was 1 mV·s^−1^. The *η*_PDP_ values were calculated as follows:

(2) $$\eta_{\mathrm{PDP}}(\%)=\frac{i_{\mathrm{corr},0}-i_{\mathrm{corr}}}{i_{\mathrm{corr},0}}\times 100$$

The *i*_corr_ and *i*_corr,0_ are the current density of steel with and without ATL extract. All electrochemical experiments were conducted three times to ensure repeatability.

### 2.4. Weight Loss and Morphology Analysis

The experimental standard of weight loss was ASTM G31-21 (USA) [20]. Before immersing in corrosive solutions, the steel sample was polished (220 to 2000 mesh), cleaned (99.5 wt.% EtOH), and dried (N_2_). After that, it was weighed by a high-precision electronic balance and immersed in a corrosive solution with different concentrations of ATL extract at 298 K. Following the 24 h immersion, the sample was ultrasonically washed, dried, and weighed again. Two equations were employed to collect the corrosive rate (*v*) and *η*_w_ [26].

(3) $$\nu =\frac{\Delta W}{\mathrm{At}}$$

(4) $$\eta_{W}\left(\%\right)=\frac{\nu_{0}-\nu }{\nu_{0}}\times 100$$

where Δ*W* is the corrosive loss of the same sample, *A* stands for the soaking area, and *t* represents the experimental time. *v* and *v*_0_ mean the corrosive rates of Q235 steel with and without ATL extract.

The specimen utilized for morphological examination was polished to a fineness of 7000 mesh and subsequently cleansed to eliminate any oily residue. Next, the sample was submerged in 1 M HCl solution with and without 1000 mg/L ATL extract for 24 h. After that, SEM/EDS (Regulus 8230, HITACHI, Tokyo, Japan) and LSCM (LSM 900 ZEISS, Jena, Germany) were utilized to examine the morphology and roughness alteration of the steel sample [27]. The SEM/EDS test was conducted using secondary electron mode, with a vacuum level of 10^−6^ Pa. The potential acceleration was set at 15 kV, the working distance was 6.2 mm, and the electron gun used was ZrO/W. For the LSCM, the test condition was room temperature, and the scan area was 106.5 × 106.5 μm. The diode laser was 405 nm (5 mW), and the objective lens was 63 × oil (NA1.4).

### 2.5. Calculation Details

The DFT calculation was employed to analyze the interface interaction between ATL extract molecules (syringic acid (SA), quercetin (QU), and chlorogenic acid (CA)) and the Q235 steel surface [28]. The simulative software was Gaussian 09 W, and the geometric optimization of three ATL extract molecules was performed via the 6-31G(d) module with the RB3LYP method [29]. The computation was finished in the gaseous environment. Notably, a series of critical parameters were collected and discussed carefully, such as the highest occupied molecular orbital (*E*_HOMO_), the lowest unoccupied molecular orbital (*E*_LUMO_), and the dipole moment (*μ*).

The adsorption state and binding energy (*E*_binding_) of ATL extract molecules on the steel (110) crystal face were simulated via MD simulation. The simulated cell size was 25.6 × 21.8 × 60.8 Å, and an extra 30 Å vacuum layer was included [30]. Significantly, the simulated cell contained one ATL extract molecule (SA, QU, or CA) and 500 water molecules, which conformed to the COMPASSⅢ force field. A NVT canonical ensemble and the Nose-Hoover method were used to ensure the computational precision and temperature (298.15 K) [31]. The molecular structure was output once per 500 steps, with a total computation time of 1000 ps and a time step of 1 fs.

## 3. Results and Discussion

### 3.1. The Chemical Ingredient of ATL Extract

**Figure 2:**
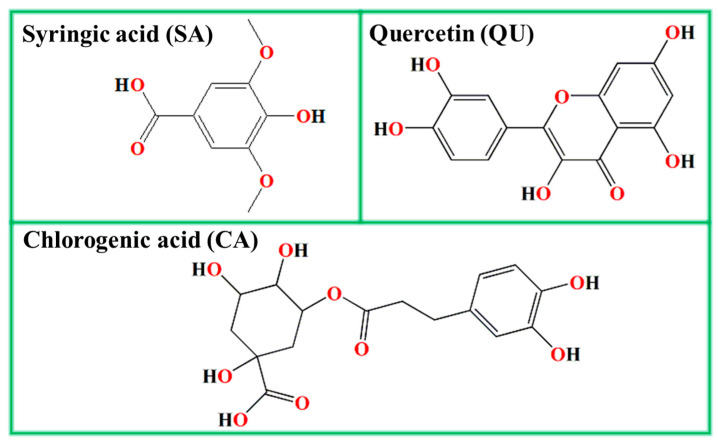
The leading organic ingredients of ATL extract.

**Figure 3:**
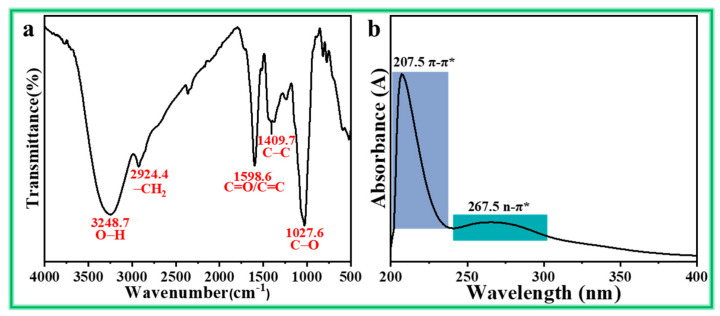
The (**a**) FT-IR and (**b**) UV−Vis spectra of ATL extract.

As shown in Figure 2, the leading organic ingredients of ATL extract include syringic acid, quercetin, and chlorogenic acid, which is also proved by liquid chromatography tandem-mass spectrometry in the work of Sucur et al. [25]. The FT-IR and UV-Vis spectra of the ATL extract are given in Figure 3 to verify its primary functional groups. For the FT-IR spectrum, the stretching vibration of phenolic hydroxyl groups in syringic acid, quercetin, and chlorogenic acid is presented at 3248.7 cm^−1^. The stretching vibration of the -CH_2_ bond that comes from the carbon chain is noticed at 2924.4 cm^−1^. The C=O/C=C stretching vibration peak of carboxide/carboxyl and the unsaturated double-bond is located at 1598.6 cm^−1^. The distinctive absorption of benzene rings is present at 1409.7 cm^−1^. The absorption peak of C-O is detected at 1027.6 cm^−1^ [32].

As depicted in Figure 3b, two apparent absorption peaks are observed in the UV-Vis spectrum of ATL extract. The absorption peak at 267.5 nm corresponds to the n-π* transition of C=O in carboxide/carboxyl, while the peak at 207.5 nm represents the π-π* transition of the benzene ring skeleton structure [33]. Undoubtedly, the ATL extract contains a significant amount of carbonyl groups, unsaturated double bonds, heteroatoms, and aromatic rings. These components are advantageous for creating a dense and stable inhibition film on the steel surface, thereby reducing its corrosion rate.

### 3.2. DFT Calculations

**Figure 4:**
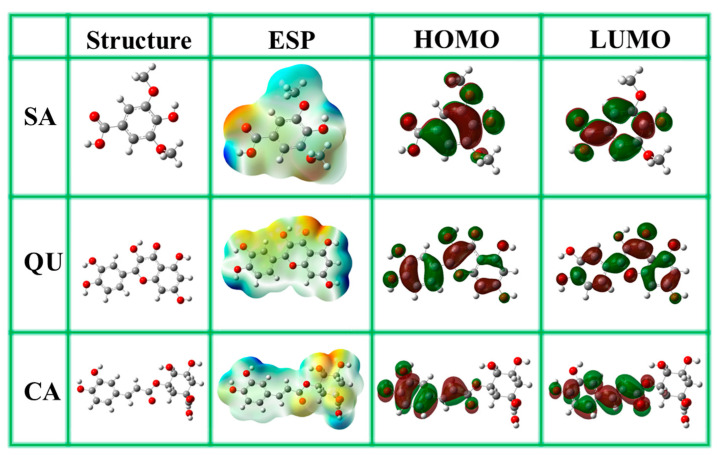
The structure, ESP, HOMO, and LUMO of SA, QU, and CA in ATL extract.

As displayed in Figure 4, the optimized molecular structures of SA, QU, and CA are coplanar, which may facilitate their parallel adsorption on the steel surface. ESP mapping (electrostatic potential) shows that the negative (blue) regions in connection with nucleophilic reactivity are distributed on benzene rings and O atoms. Simultaneously, the electron clouds of the HOMO and LUMO are evenly distributed within each molecule, particularly in carboxide, carboxyl, the benzene ring, and unsaturated double bonds. Based on these observed behaviors, the presence of these active functional groups can increase the competitiveness of ATL extract molecules and boost the speed and durability of their adsorption on steel surfaces. The HOMO/LUMO electron cloud is not distributed on its benzene ring for the CA molecule, possibly due to the substantial steric hindrance.

In general, *E*_HOMO_ means the electron-donating ability, while the *E*_LUMO_ is the electron-receiving property. The inhibitor with high *E*_HOMO_ and low *E*_LUMO_ values can be readily adsorbed to metal surfaces [34]. In this work, the *E*_HOMO_/*E*_LUMO_ values of SA, QU, and CA are −6.06/−1.22 eV, −5.47/−1.73 eV, and −5.82/−1.81 eV, respectively. A smaller energy gap (Δ*E* = *E*_LUMO_−*E*_HOMO_) represents the robust adsorption performance of inhibitor molecules [35]. The Δ*E* values of SA (4.84 eV), QU (3.74 eV), and CA (4.01 eV) are calculated based on their *E*_HOMO_ and *E*_LUMO_. Notably, the dipole moment (*μ*) is an essential parameter in estimating the adsorption capacity of inhibitors, and a higher *μ* value often indicates a more robust adsorption capacity [36]. The *μ* values of SA, QU, and CA are 3.1D, 8.2 D, and 4.2 D. Clearly, all quantum chemical calculations show an identical outcome. The extractant molecules have intense adsorption activity and may build an efficient protective film on the steel surface.

**Figure 5:**
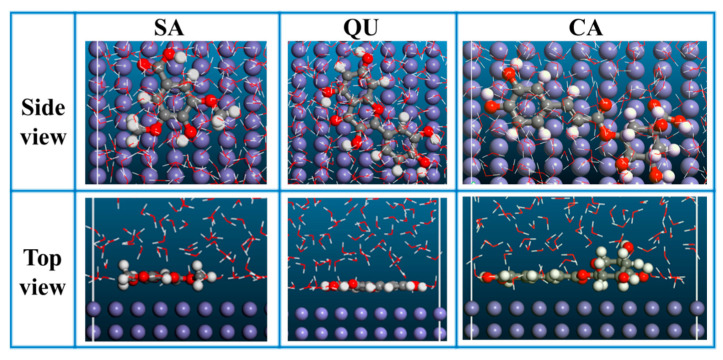
Equilibrium adsorption configuration of SA, QU, and CA molecules on Fe (110) crystal face.

The adsorbed states of three ATL extract molecules on Fe (110) are depicted in Figure 5. The side and top views clearly show that SA, QU, and CA molecules are adsorbed in a parallel structure on Fe (110). This phenomenon demonstrates that ATL extract molecules have the ability to obstruct the contact between the metal surface and the corrosive solution. Meanwhile, it also indicates that ATL extract molecules are superior in competitive adsorption with corrosive media. Strong adsorption interaction and remarkable inhibition efficacy have been demonstrated to correlate with a high *E*_binding_ value between the inhibitor and the metal [37]. The obtained *E*_binding_ values are 157.90 kcal/mol for SA, 222.94 kcal/mol for QU, and 259.66 kcal/mol for CA, which may indicate that ATL extract molecules can provide high-efficiency protection for steel in hydrochloric acid.

### 3.3. EIS Analysis

**Figure 6:**
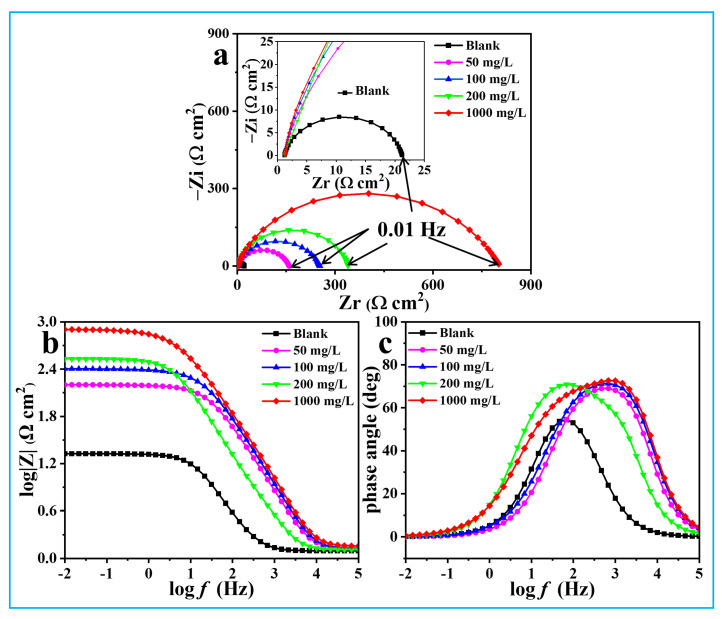
The Nyquist (**a**), Impedance modulus (**b**), and phase angle (**c**) diagrams of Q235 steel in 1 M HCl solution with various concentrations of ATL extract.

EIS, a quick and nondestructive test technology, is often used to assess the protective efficacy of plant extract inhibitors on steel substrates in acidic environments. The EIS spectra of Q235 steel with and without the protection of ATL extract are given in Figure 6. The Nyquist spectrum (Figure 6a) of steel in bulk solution exhibits a depressed semi-loop (capacitive loop), which is caused by double-layer capacitance and charge transfer resistance. The centers of these depressed loops are displaced below the real axis. This phenomenon may be related to the frequency dispersion of the interfacial impedance and the inhomogeneous steel surface because of the microscopic roughness and inhibitor adsorption [38]. The addition of ATL increases the size of the impedance plots, suggesting that the organic compounds in the ATL form a protective film on the surface of the Q235 steel. The capacitive loop increases with increasing inhibitor concentration, as many more inhibitor molecules adsorb on the steel surface, indicating that the inhibitor film gradually becomes compact and ultimately leads to an improved protective effect [39]. The Nyquist spectra obtained with ATL extract closely resemble the blank spectrum, suggesting that the corrosion mechanism remains unchanged and is still governed by charge transfer.

As illustrated in Figure 6b,c, relative to the blank, the width and height of the phase angle and the impedance modulus increase visibly with the presence of the corrosion inhibitor. Moreover, there is a linear relationship between logZ and logf with a slope near −1, proving that ATL forms a dense film on copper. These results confirm the superior inhibition ability of ATL extract.

**Figure 7:**
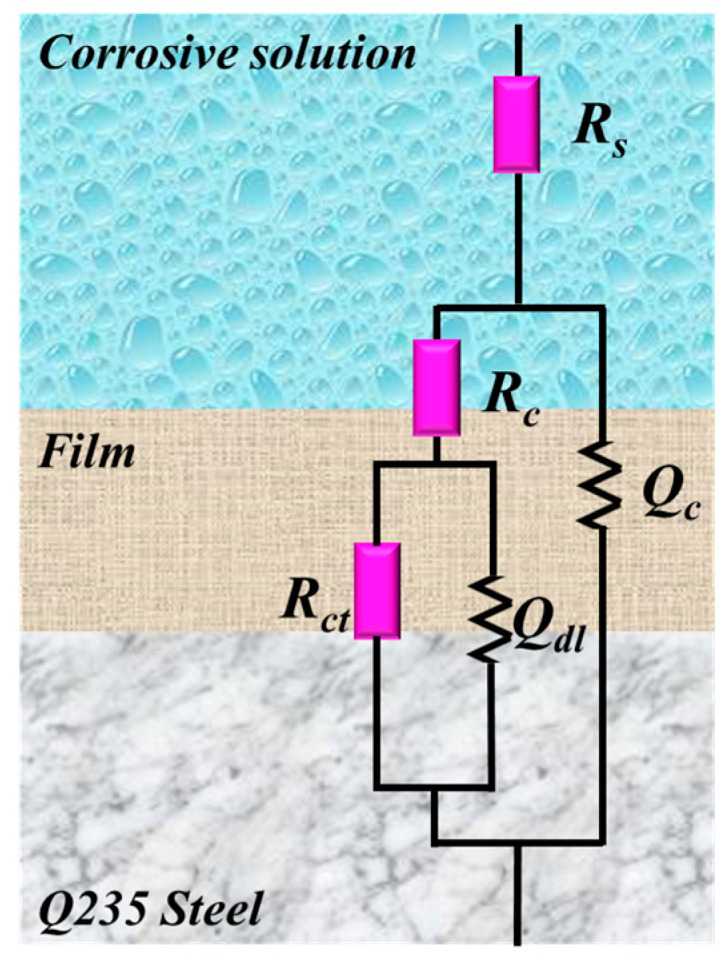
The suitable equivalent circuit used to fit EIS data.

The high-frequency peaks are clearly visible, caused by the electric double-layer capacitance. However, the characteristic frequency peaks belonging to the film capacitor are not pronounced. In addition, the highest frequency platforms from Figure 6b correspond to the solution resistance, and the lowest frequency platforms represent the total resistance. Therefore, the EIS data is fitted using a suitable equivalent circuit (Figure 7), considering the multiple interface relationships between Q235 steel, ATL extract, and acid solution. The χ^2^ values of all EIS data consistently remain at the 10^−4^ level, indicating the reliability of the equivalent circuit. A series of relevant fitted parameters, including *R*_s_ (solution resistance), *R*_f_ (film resistance), *R*_ct_ (charge transfer resistance), *C*_f_ (film capacitance), and *C*_dl_ (double electrode layer capacitance), are obtained and displayed in Table 1. Notably, *C*_f_ and *C*_dl_ are calculated as follows [40]:

(5) $$C=Y_{0}{\left(\omega \right)}^{n-1}=Y_{0}{(2\pi f_{Z_{\mathrm{im}-\mathrm{Max}}})}^{n-1}$$

*Y*_0_ is the CPE constant, *ω* means the angular frequency, n represents the deviation parameter, and $f_{Z_{\mathrm{im}-\mathrm{Max}}}$ stands for the frequency corresponding to the max imaginary part on the impedance spectrum.

The values in Table 1 show a decrease in *C*_f_ and *C*_dl_, while *R*_f_ and *R*_ct_ values increase with the addition of ATL extract. This trend becomes even more noticeable as the ATL extract concentration is enhanced. The interaction between steel and acid solution is slowed down by the presence of ATL extract, especially at a higher ATL concentration. Essentially, a competitive adsorption relationship exists between ATL extract molecules and corrosive media on the steel surface. However, benefiting from unsaturated double bonds, aromatic rings, and carbonyl groups, ATL extract molecules can readily adsorb on the steel surface and create an inhibition film, as proved by theoretical calculations [41]. Notably, the *R*_ct_ value of steel in bulk acidic solution is 17.1 Ω cm^2^, but it significantly increases to 765.0 Ω cm^2^ when the additive amount of ATL extract is 1000 mg/L. In contrast, the *C*_dl_ values decrease significantly from 134.0 μF cm^−2^ (bulk solution) to 1.5 μF cm^−2^ (1000 mg/L ATL). Additionally, the *η* value increases as the concentration of ATL extract rises, ultimately reaching an impressive 97.5%. In conclusion, the ATL extract demonstrates remarkable inhibition properties for Q235 steel corrosion in acidic solutions.

Table 2 shows other reported plant extracts as steel corrosion inhibitors in the HCl acid. Regretfully, some extracts have higher added dosages than ATL extract, but their inhibition ability is lower. To summarize, a small dose of ATL extract can offer superior inhibition for steel in a harsh acid solution.

**Figure 8:**
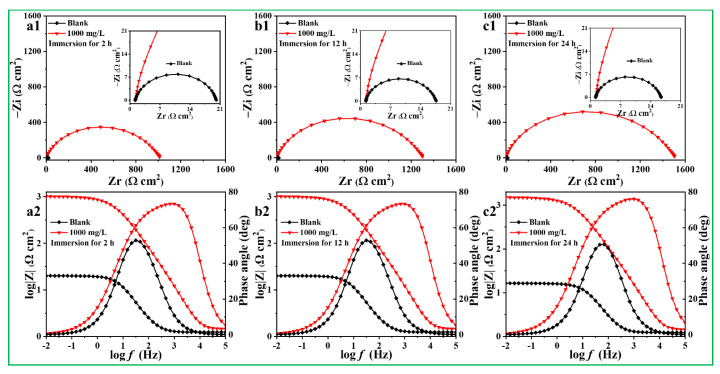
The Impedance spectra of steel without and with 1000 mg/L ATL extract in different immersion times at 298 K: (**a1**,**a2**) 2 h, (**b1**,**b2**) 12 h, and (**c1**,**c2**) 24 h.

It is important to note that the time factor should also be considered when evaluating the protection property of inhibitors for the metal in corrosive solutions [48]. Therefore, the impedance spectra of Q235 steel immersed in acidic solutions, with and without 1000 mg/L ATL extract for 24 h, are presented in Figure 8. There are two distinct stages in the corrosive process of steel in HCl solution over the immersion time. The Nyquist spectrum shows a noticeable reduction after the initial 12 h immersion, suggesting the steel has undergone severe corrosion. As the immersion period is increased to 24 h, the capacitive arc size of steel tends to remain stable, indicating that the corrosion of steel is irreversible [49].

It is worth noting that the Nyquist curve of steel in the presence of 1000 mg/L ATL extract is more extensive compared to the blank. In addition, the radius of the Nyquist spectrum consistently grows over time, indicating the enhanced corrosion inhibition of ATL extract for steel. Interestingly, the height and width of the impedance modulus and the phase angle noticeably enhance when the immersing duration is extended. The findings confirm the reliability of the protective film, which becomes more compact with a longer immersion time [50]. The EIS curves have been fitted by the equivalent circuit in Figure 7, and related impedance parameters are presented in Table 3. Upon completion of the 24 h immersion, the *R*_ct_ values of steel with 1000 mg/L ATL extract are 1462.0 Ω cm^2^, which is 115.1 times higher than those of blank (12.7 Ω cm^2^). At the same time, the *η* value of 1000 mg/L ATL extract demonstrates an improved trend, increasing from 97.5% to 99.0%. These observations illustrate that ATL extract can offer sustained protection for steel in acidic environments, even after prolonged periods of immersion.

### 3.4. Polarization Curve Analysis

**Figure 9:**
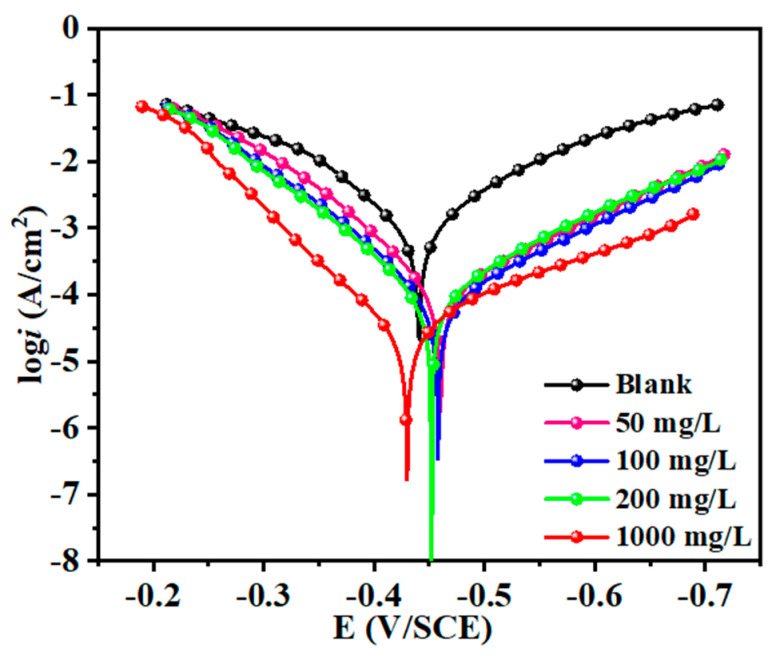
The polarization curves of Q235 steel in 1 M HCl with various concentrations of ATL extract.

As given in Figure 9, the cathodic and anodic branches of Q235 steel exhibit a noticeable downward shift when the ATL extract is added, and this effect becomes more pronounced as the concentration increases. This discovery suggests that the ATL extract can inhibit both the cathodic and anodic corrosion reactions, particularly when higher doses of the ATL extract are presented. It is essential to mention that the shape of all cathodic and anodic branches is similar, indicating that the reaction mechanism is consistent. The hydrogen evolution and steel dissolution reactions still govern the cathode and anode, showing that ATL extract only reduces the reaction rate [51].

Typically, the Tafel region of the potentiodynamic polarization curve exhibits a linear curve. The corrosion current density and Tafel slope can be calculated by extrapolating the linear part of the Tafel line (extrapolation approach). As presented in Table 4, several significant polarization properties are determined via the extrapolation in the Tafel area (>±118 mV Vs. corrosion potential, E_corr_), such as the corrosion current density (*I*_corr_) and branch slopes (*β*_c_/*β*_a_). The addition of ATL extract reduces the *I*_corr_, and this effect gets more distinct as the concentration increases. At 1000 mg/L ATL extract concentration, the *I*_corr_ value is lower (29.4 μA cm^–2^) than the blank (1101.0 μA cm^–2^), showing a substantial reduction of approximately 37.4 times. Furthermore, the *η*_PDP_ value significantly increases when combined with ATL extract, reaching an impressive 97.3%. The slight fluctuation in *β*_c_ and *β*_a_ validates the consistency of cathodic and anodic reaction processes. The maximum fluctuation observed in all *E*_corr_ values is less than 85 mV (precisely 19 mV), indicating that the ATL extract functions as a mix-type inhibitor. These results demonstrate that ATL extract can create a protective film that effectively restrains the cathodic and anodic processes of steel in harsh acidic solutions.

### 3.5. Immersion Test and Adsorption Type

**Figure 10:**
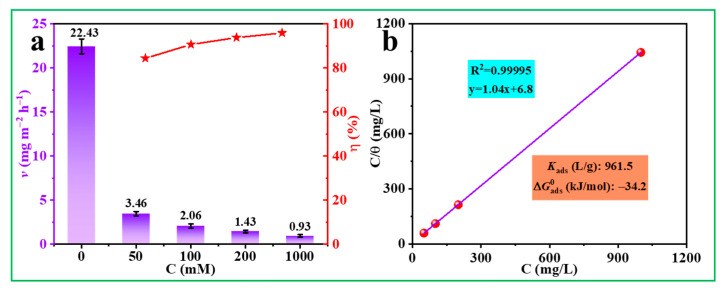
(**a**) The corrosive rate and protection property of different ATL concentrations and (**b**) relevant adsorption model.

The immersion experiment is conducted at 298 K to determine the long-term and comprehensive corrosion rate of the Q235 steel in acidic solutions. According to Figure 10a, the *v* value of steel without ATL extract is the highest (22.43 mg m^−2^ h^−1^). The inclusion of ATL extract (50 mg/L) causes a rapid decrease in *v* value to 3.46 mg m^−2^ h^−1^, which steadily declines to 0.93 mg m^−2^ h^−1^ as ATL extract concentration increases to 1000 mg/L. Additionally, there is a reinforcing trend in the inhibition capacity, with the highest value of 95.9% (1000 mg/L), confirming the long-term and reliable protection of ATL extract for Q235 steel in line with the electrochemical data.

The weight loss data is carefully analyzed to explore the adsorption type of ATL extract. Various adsorption models, including Fumkin, Floy-Huggins, Langmuir, and others, are employed to study the interaction at the interface between metal and plant extract inhibitors. Figure 10b shows that the coefficient of association is 0.99995, which suggests a feasibility and linear relationship between the weight loss data and the Langmuir adsorption isotherm. Hence, this model is the most suitable for explaining the interaction between steel and ATL extract molecules at the interface. Under this circumstance, ATL extract molecules can rapidly adsorb on the metal substrate, forming a dense monolayer protection film that effectively reduces the steel corrosion rate. The following equations are used to calculate and thoroughly discuss two crucial thermodynamic parameters: equilibrium (*K*_ads_) and standard adsorption-free energy (Δ$G_{\mathrm{ads}}^{0}$) [52].

(6) $$\frac{C}{\theta }=\frac{1}{K_{\mathrm{ads}}}+C$$

(7) $$K_{\mathrm{ads}}=\frac{1}{1000}\exp(-\frac{\Delta G_{\mathrm{ads}}^{0}}{\mathrm{RT}})$$

where *C* denotes the concentration of ATL extract, *θ* signifies steel surface coverage, *R* represents the molar gas constant, and *T* corresponds to the Kelvin temperature.

As seen in Figure 10b, the *K*_ads_ value (961.5 L/g) is relatively high, suggesting that the molecules of ATL extract have a strong ability to adsorb steel in acidic solutions. It is widely recognized that absorption can be divided into chemisorption (Δ$G_{\mathrm{ads}}^{0}$ ≤ −40 kJ/mol) and physisorption (Δ$G_{\mathrm{ads}}^{0}$ ≥ −20 kJ/mol) [53]. In this work, the Δ$G_{\mathrm{ads}}^{0}$ value (−34.2 kJ/mol) demonstrates a protection film assembled on the steel surface via mix-type adsorption, further obstructing the contact between the acidic solution and steel substrate. In addition, the Δ$G_{\mathrm{ads}}^{0}$ value near −40 kJ/mol indicates that the film-forming process is primarily governed by chemisorption, with physisorption playing a supporting role.

### 3.6. Morphology Observation

**Figure 11:**
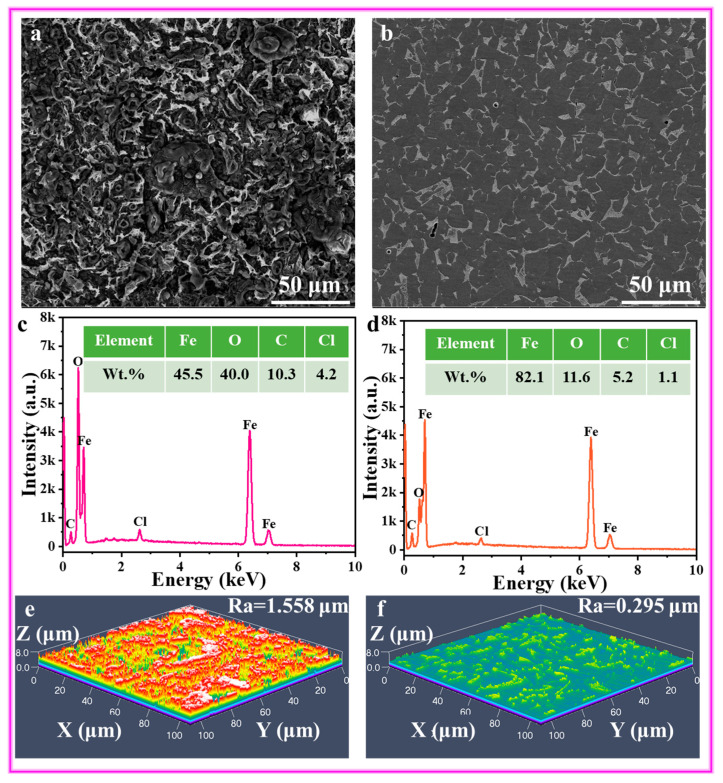
SEM/EDS (**a**–**d**) and LSCM (**e**,**f**) plots of Q235 steel without and with 1000 mg/L ATL extract after immersion 24 h.

Figure 11 displays the SEM and LSCM images of the Q235 steel after 24 h of immersion with 1000 mg/L ATL extract. The surface morphology of steel is relatively rough, with a large amount of flocculent and circle-shaped corrosion products, demonstrating that bare steel is hard to resist acid corrosion during long-term immersion. Meanwhile, the corrosion morphology exhibits heterogeneity, possibly influenced by the lattice type and surface roughness. In Figure 11b, under the action of 1000 mg/L ATL extract, the surface morphology of steel is clean and tidy. Besides artificial scratches, only mild local corrosion behavior is observed along the entire surface. As observed in the EDS results, the weight ratio of Fe, O, and Cl elements in pure acidic solution is 45.5 wt.%, 40.0 wt.%, and 4.2 wt.%, while the value with ATL extract is 82.1 wt.%, 11.6 wt.%, and 1.1 wt.%, respectively. The results demonstrate that ATL extract can create a compact and robust organic film, resulting in outstanding and consistent protection for Q235 steel in acidic solutions.

As displayed in Figure 11c, the 3D morphology of the metal without ATL extract is rough, exhibiting numerous ridges and ravines. Simultaneously, the *R*_a_ value reaches as high as 1.558 μm, which means bare steel is highly susceptible to damage from the corrosive media. In Figure 11d, it can be observed that adding 1000 mg/L ATL extract results in a smooth steel surface with only a minimal accumulation of corrosion products. Furthermore, the *R*_a_ value decreases to 0.295 μm, significantly smaller than the blank. These findings agree with electrochemical and SEM analysis results, indicating that the ATL extract effectively inhibits Q235 steel in 1 M HCl solutions.

### 3.7. Inhibition Mechanism

To explain the interaction at the ATL extract/steel interface, offer valuable experience for future investigations, and promote the industrial application of plant extract inhibitors, an insightful discussion of the interaction at the ATL extract/steel interface in acidic solutions is presented. Notably, the initial occurrence is the anodic dissolution reaction of Q235 steel, as follows:

(8) $$\mathrm{Fe}+H_{2}O\to \mathrm{Fe}(\mathrm{OH}{)}_{\mathrm{ads}}+H^{+}+e^{-}$$

(9) $$\mathrm{Fe}(\mathrm{OH}{)}_{\mathrm{ads}}\to \mathrm{Fe}(\mathrm{OH}{)}^{+}+e^{-}$$

(10) $$\mathrm{Fe}(\mathrm{OH}{)}^{+}+H^{+}\to Fe^{2+}+H_{2}O$$

After that, the cathode region of steel experiences a hydrogen evolution reaction as a result of charge transfer.

(11) $$\mathrm{Fe}+H^{+}\to (\mathrm{Fe}H^{+}{)}_{\mathrm{ads}}$$

(12) $$(\mathrm{Fe}H^{+}{)}_{\mathrm{ads}}+e^{-}\to (\mathrm{FeH}{)}_{\mathrm{ads}}$$

(13) $$(\mathrm{FeH}{)}_{\mathrm{ads}}+H^{+}+e^{-}\to \mathrm{Fe}+H_{2}↑$$

**Figure 12:**
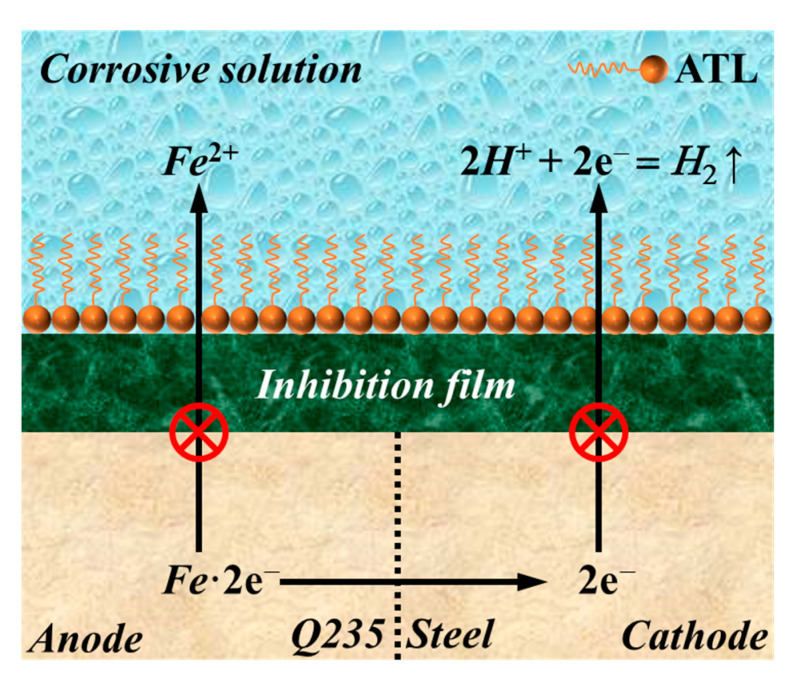
The inhibition mechanism of ATL extract for Q235 steel in corrosive solution.

In this environment, steel suffers from severe corrosion. As depicted in Figure 12, a compact and robust inhibition film can be rapidly formed on the steel surface through the adsorption of ATL extract. The adsorption process benefits from the interaction between ATL extract molecules and Fe^2+^, as shown in Equation (14) [54]. Throughout the adsorption process, Cl^−^ also contributes to the development of the film, gradually converting it into a remarkably durable composite protective film, as given in Equation (15) [20].

(14) $$Fe^{2+}+mH_{2}O+n\mathrm{ATL}\to [\mathrm{FeAT}L_{n}(\mathrm{OH}{)}_{m}{]}^{2-m}+mH^{+}$$

(15) $$Fe^{2+}+mH_{2}O+n\mathrm{ATL}+pCl^{-}\to [\mathrm{FeAT}L_{n}(\mathrm{OH}{)}_{m}Cl_{p}{]}^{2-m-p}+mH^{+}$$

This protective film is undoubtedly created by ferrous ions that offer vacant orbitals, while ATL extract molecules provide lone pair electrons. The protective film’s compactness and stability depend on the ATL extract concentration (active adsorption centers), which also determines its inhibitory performance. When the ATL extract is 1000 mg/L, the most stable inhibition film is formed on Q235 steel, which can significantly withstand the attack of corrosive media and exhibits excellent inhibition efficiency.

## 4. Conclusions

In this work, a green and high-efficiency ATL extract inhibitor with excellent water solubility is obtained by a deionized water extraction method. Based on advanced theoretical calculations and extensive experiments, the inhibition effects of ATL extract on Q235 steel have been thoroughly investigated for the first time. The inhibition mechanism of ATL extract molecules for the Q235 steel surface is also revealed in detail.

1. FT-IR and UV-Vis spectra show that ATL extract possesses numerous heterocyclic organics with conjugated structures. Theoretical simulations indicate that the critical active adsorption sites of ATL extract molecules include unsaturated double bonds, aromatic rings, carbonyl groups, and heteroatoms. The highest *E*_binding_ value observed for the major components in the ATL extract is 259.66 kcal/mol, implying a powerful adsorption performance.

2. Electrochemical results demonstrate that the *R*_ct_ value of steel with 1000 mg/L ATL extract (765.0 Ω·cm^2^) is 44.8 times larger than that of blank solution (17.1 Ω·cm^2^). After 24 h of immersion, the *η* value remains impressively high at 99.0%, attributed to the ATL extract film slowing down corrosion reactions of Q235 steel.

3. Weight loss experiments have demonstrated that the presence of ATL extract dramatically inhibits the dissolution rate of steel, which decreases from 22.43 to 0.93 mg m^−2^ h^−1^. The SEM/EDS and LSCM observations reveal that the ATL extract exhibits exceptional and dependable inhibition properties on steel in acidic solutions during long-term immersion.

4. The Langmuir adsorption isotherm findings demonstrate that the ATL extract spontaneously forms a protective monolayer film. This film is derived from a combination of adsorption methods, primarily chemical adsorption, which benefits from ferrous ions that offer vacant orbitals, while ATL extract molecules provide lone pair electrons.

5. The purpose of this work is to advance the practical use of novel plant extract inhibitors in industrial pickling settings by offering insights into their development.

## Figures and Tables

**Table 1:** Impedance parameters of Q235 steel with various ATL extract concentrations in acid solution at 298 K.

*C*	*R*_s_	$R_{f}^{a}$	$R_{ct}^{b}$	$C_{f}^{d}$	*n*_1_	$C_{dl}^{e}$	*n*_2_	*η*	*χ* ^2^
(mg/L)	(Ω cm^2^)	(Ω cm^2^)	(Ω cm^2^)	(μF cm^−2^)		(μF cm^−2^)		(%)	10^−4^
Blank	1.3	2.9	17.1	362.3	1	134.0	0.78	-	1.4
50	1.4	19.0	138.8	20.5	1	3.4	0.72	87.3	6.9
100	1.4	21.2	231.6	16.6	1	1.8	0.69	92.1	6.7
200	1.3	28.7	310.0	15.5	1	1.6	0.71	94.1	9.4
1000	1.4	39.0	765.0	14.4	1	1.5	0.69	97.5	6.6

^a^ The standard deviation varied between 2% and 5%. ^b^ The standard deviation varied between 3% and 7%. ^d^ The standard deviation varied between 4% and 7%. ^e^ The standard deviation varied between 2% and 6%.

**Table 2:** The previous investigations about plant extract as steel corrosion inhibitors in the HCl solution.

Inhibitor	Metal	Acid (HCl)	Concentration	*η* (%)	Reference
*E. aegyptiaca*	cast iron	1 M	2400 ppm	91.5	[32]
*Citrullus lanatus*	mild steel	1 M	2000 mg/L	83.3	[42]
*Rollinia occidentalis*	carbon steel	1 M	1000 mg/L	79.7	[43]
*Rheum Ribes*	mild steel	1 M	2000 mg/L	94.2	[44]
*Magnolia grandiflora*	Q235 steel	1 M	500 mg/L	85.5	[45]
*Randia monantha*	mild steel	1 M	1000 mg/L	91.8	[46]
*Seaweed*	carbon steel	1 M	500 mg/L	92.0	[47]
*Ambrosia trifida L*	Q235 steel	1 M	1000 mg/L	97.5	This work

**Table 3:** Impedance parameters of Q235 steel without and with 1000 mg/L ATL extract in different immersion times at 298 K.

*Time*	*C*	*R_s_*	$R_{f}^{a}$	$R_{ct}^{b}$	$C_{f}^{d}$	*n*_1_	$C_{dl}^{e}$	*n*_2_	*η*	*χ* ^2^
(h)	(mg/L)	(Ω cm^2^)	(Ω cm^2^)	(Ω cm^2^)	(μF cm^−2^)		(μF cm^−2^)		(%)	10^−4^
2	0	1.3	2.5	16.4	366.4	1	158.5	0.69	-	8.4
	1000	1.4	37.9	978.0	12.0	1	0.96	0.67	98.1	4.7
12	0	1.3	2.2	14.2	441.8	1	170.4	0.72	-	1.7
	1000	1.4	43.4	1265.0	10.9	1	0.79	0.67	98.7	7.7
24	0	1.2	2.6	12.7	507.4	1	176.6	0.73	-	7.2
	1000	1.4	51.4	1462.0	0.94	1	0.57	0.67	99.0	7.0

^a^ The standard deviation varied between 3% and 7%. ^b^ The standard deviation varied between 4% and 8%. ^d^ The standard deviation varied between 3% and 5%. ^e^ The standard deviation varied between 4% and 6%.

**Table 4:** The polarization parameters of steel with various ATL extract concentrations in acid solution at 298 K.

*C* (mg/L)	$E_{corr}^{a}$ (V/SCE)	$I_{corr}^{b}$ (μA cm^–2^)	$\beta_{c}^{c}$ (mV dec^–1^ )	$\beta_{a}^{d}$ (mV dec^–1^ )	*η* (%)
Blank	−0.441	1101.0	−115.4	95.5	-
50	−0.460	123.8	−122.1	73.9	88.8
100	−0.458	82.8	−120.8	75.1	92.5
200	−0.452	68.3	−111.5	77.1	93.8
1000	−0.430	29.4	−152.9	64.1	97.3

^a^ The standard deviation varied between 3% and 5%. ^b^ The standard deviation varied between 3% and 5%. ^c^ The standard deviation varied between 2% and 4%. ^d^ The standard deviation varied between 3% and 7%.

## Data Availability

All data contained within the article.
